# A multiplex Taqman PCR assay for MRSA detection from whole blood

**DOI:** 10.1371/journal.pone.0294782

**Published:** 2023-11-27

**Authors:** Suhanya Duraiswamy, Sushama Agarwalla, Khoi Sheng Lok, Yee Yung Tse, Ruige Wu, Zhiping Wang

**Affiliations:** 1 Department of Chemical Engineering, Indian Institute of Technology Hyderabad, Telangana, India; 2 Singapore Institute of Manufacturing Technology (SIMTech), Agency for Science, Technology and Research (A*STAR), Singapore, Republic of Singapore; Tribhuvan University, NEPAL

## Abstract

Methicillin-resistant *Staphylococcus aureus* (*MRSA*) causes a wide range of hospital and community-acquired infections worldwide. *MRSA* is associated with worse clinical outcomes that can lead to multiple organ failure, septic shock, and death, making timely diagnosis of *MRSA* infections very crucial. In the present work, we develop a method that enables the positive enrichment of bacteria from spiked whole blood using protein coated magnetic beads, followed by their lysis, and detection by a real-time multiplex PCR directly. The assay targeted bacterial 16S rRNA, *S*. *aureus* (*spa*) and methicillin resistance (*mecA*). In addition, an internal control (lambda phage) was added to determine the assay’s true negative. To validate this assay, staphylococcal and non-staphylococcal bacterial strains were used. The three-markers used in this study were detected as expected by monomicrobial and poly-microbial models of the *S*. *aureus* and coagulase-negative staphylococci (*CoNS*). The thermal cycling completed within 30 mins, delivering 100% specificity. The detection LoD of the pre-processing step was ∼ 1 CFU/mL from 2-5mL of whole blood and that of PCR was ∼ 1pg of NA. However, the combined protocol led to a lower detection limit of 100–1000 *MRSA* CFUs/mL. The main issue with the method developed is in the pre-processing of blood which will be the subject of our future study.

## Introduction

Viable bacteria or fungi in blood stream causes blood stream infections (BSI) that pose significant healthcare problems. Late or improper diagnosis of BSI can result in sepsis, a life-threatening condition characterized by sudden influx of chemicals into the bloodstream which leads to multiple organ failure i.e., organs lose their regular functional capabilities resulting ultimately in death [1]. Diagnosis and treatment of BSI typically involves collecting blood from patients and processing them to detect and identify pathogen causing BSI, followed by treating patients with common antibiotics. *Staphylococcus aureus* (SA) a gram-positive, round-shaped bacterium, frequently found in nose, respiratory tract, and on skin is a common cause of BSI [1] and the emergence of drug-resistant strains such as *Methicillin Resistant* SA (*MRSA*), is now a major concern [2]. *MRSAs* were historically treatable with common antibiotics but have developed resistance to various beta-lactam antibiotics (sub-types oxacillin and methicillin) as well as to other antimicrobial classes such as aminoglycosides, carbapenems, cephalosporins, fluoroquinolones and macrolide; this is typically triggered by natural selection or through horizontal gene transfer. The staphylococcal cassette chromosome mec (SCCmec), which has at least 13 different forms, contains mecA, a mobile gene element is responsible for resistance. Most of the resistance is secondary to the production of beta-lactamase enzymes or intrinsic resistance with alterations in penicillin-binding proteins, PBP2a, which is encoded in the *mecA* gene [3–5]. Though MRSAs are not more virulent than *methicillin-sensitive SA* (*MSSA*), they definitely pose severe treatment challenges and are reported to lead to worse clinical outcomes, greater healthcare costs and mortality [6, 7].

Identifying the *mecA* gene using polymerase chain reaction (PCR) assays is now widely accepted as the strategy for detection of *MRSA*. However the *mecA* gene is also harboured by *coagulase-negative Staphylococcus* (CoNS) and are often misidentified as *MRSA* [8]. *CoNS* is negative for coagulase but positive for catalase unlike SA that is coagulase positive, a virulence factor. A coagulase test is hence carried out to identify the coagulase-positive strain of SA, if the sample is positive for catalase. Additionally, such diagnostic tools using PCRs to detect pathogen harbouring the *mecA* gene depend heavily on the pre-processing of patient blood to improve the selectivity and sensitivity of the assay. Positive enrichment of pathogen from blood involves isolating pathogen selectively from blood and is considered for developing many diagnostic tools [9–11], which is also a novelty in our current work.

Numerous researchers have reported multiplex PCR assays, which accurately detect the *mecA* gene, correlating well with the oxacillin-resistant phenotype [12–14]. Among these methods, real-time multiplex PCR (RT-PCR) offers the shortest assay time, lowest detection limit and highest sensitivity and specificity. Identification of *MRSA* directly using *SCCmec*-*orfx* element was performed by Huletsky *et al*.[15]. Commercial assays implemented this concept using SCC, which often misidentified *MRSA* due to the absence of *CoNS* detection protocol [16, 17]. To tackle this, PCR assays for species identification of *CoNS* (using additional markers such as *coa*, *nuc*, *spa* or *femA*) were developed, though not housed in a single reaction and were incomplete [18–21]. An all-in-one heptaplex PCR assay for identification of SA and *CoNS* was developed by Okolie *et al*. [22, 23], which can simultaneously detect virulence and antibiotic resistance genes. However, the former was only an end point detection via gel electrophoresis and the latter did not have a positive control thus being unable to distinguish assay failure from true negative. There is hence a critical need to have a discriminatory assay that can identify the cohabitation of SA and *CoNS*.

In our current work, we introduce a triplex PCR assay which addresses the need for a discriminative assay to specifically identify *MRSA* and we also integrate it with the pre-processing step that would enhance the quality of the DNA sample for MRSA identification. Hence our 2-pronged approach in this work involves using protein-coated magnetic beads for the direct capture of pathogen selectively from whole blood followed by its lysis using a sonicator horn and further processing it through the developed multiplex Taqman PCR assay for rapid detection of *MRSA*. This is one among the first to investigate the possibility of performing a direct PCR detection using spiked blood without first performing a blood culture step.

## Materials and methods

### Bacterial culture

Bacterial strains were obtained from American Type Culture Collection (ATCC, USA) or Deutsche Sammlung von Mikroorganismen und Zellkulturen GmbH (DSMZ). They were cultured using standard protocols [24]. To test for specificity, a collection of human-associated *CoNS* including strains of *Staphylococcus capitis*, *Staphylococcus saprophyticus*, *Staphylococcus epidermidis*, *Staphylococcus haemolyticus* and *Staphylococcus warneri* were used. Coagulase-positive *staphylococci* (*CoPS*) including strains of *Staphylococcus pseudintermeidus*, *SA* were also tested. Non-staphylococcal bacterial strains of diverse backgrounds including strains of *Acetobacter baumannii*, *Bacillus subtilis*, *Burkholderia cepacia*, *Candida spp*, *Enterobacter spp*, *Enterococcus spp*, *Escherichia coli*, *Klebsiella spp*, *Morganella morganii*, *Pseudomonas spp*, *Proteus mirabilis*, *Pseudomonas aeruginosa*, and *Streptococcus spp* were also studied. Target bacterial gene used in this study are general bacteria (DH5α, 16s rRNA), *Methicillin-resist (mecA)*, *S*. *aureus (Spa)*, *Positive control (Lambda dna* (specified as Ldna henceforth)).

The required bacterial concentration (the actual concentration was measured using titration on agar plates) was spiked in rabbit’s blood to simulate the cases of BSI patient’s sample (∼10 to 10^4^ CFU/mL).

### Isolation of bacteria

The spiked bacterial samples were extracted from ApoH-CaptoBAC beads kit (ApoH Technologies, France). Manufacturer protocol in brief includes the addition of equal volumes of 2X TTGB buffer (∼ 1 mL), to the collected spiked sample (∼ 1 mL) and 20 μL of ApoH beads. Then, the mixture was incubated for 30 min at 37°C in a thermomixer maintained at 1000 rpm. The reaction tube was then placed onto a magnetic stand until all ApoH magnetic beads were laterally pelleted. The pellet containing the beads were then dispersed in 100 μL 1X PBS and the supernatant blood was collected separately. The beads, supernatant blood and the original spiked blood were spread onto agar plates to determine of pathogen concentration in each of them. This experiment was repeated 5 times.

### Thermal lysis

The frozen culture was thawed and centrifuged at 13,000 rpm for 5min and the supernatant was removed. The pellet was re-suspended and washed in phosphate buffer saline (PBS), followed by centrifugation and re-suspension in ultra-pure water (Merck Millipore, US). The sample was then heated in the thermos-block at 95°C for 20 min, and immediately cooled on ice prior to centrifugation. The supernatant, which contains the gDNA, was transferred to a new microtube and used for subsequent PCR specificity study.

### Lysis using sonicator horn

A commercial sonication horn (KITVC1044, Sonics and Materials) was used to lyse the captured bacteria after pathogen isolation with ApoH-CaptoBAC beads kit. PCR was done directly with the lysate. Further details are provided in our earlier study [25].

### Qiagen kit for DNA extraction

The bacterial genomic DNA (gDNA) were purified using a Qiagen DNA extraction kit (DNeasy Blood & Tissue kit (Qiagen, Netherlands)), using the manufacturer’s recommended protocol [26]. The DNA concentration was measured using Nanodrop 2000 spectrometer (Thermofisher Scientific, USA).

### Design of oligonucleotide primers and Taqman probes

The synthesized and modified oligonucleotides were purchased from Bio Basic Canada and Integrated DNA Technologies (IDT). The oligonucleotides specifications are listed in Table 1. In brief, there are five sets of primers, utilising the five different channels of the real-time thermocycler. To design a set of universal primers that can amplify segment of 16s rRNA across the bacterial domain, the forward primer, Taqman probe, and reverse primer need to be located on the conserved region in tandem. The 16s rRNA gene sequences were downloaded from National Center for Biotechnology Information (NCBI) genome database (www.ncbi.nlm.nih.gov/). The gene sequences were aligned using clustalW multiple alignments with the aid of MEGA7 software [27]. The annealing *T*_*m*_ and the primer quality were checked using fastPCR software, Primer Digital. High linguistic complexity, >80%, and high primer’s PCR Efficiency, >80%, primers were selected for use. Three sets of 16s primers were designed for this study (see Table 1). FAM_F1, 2 and 3 used B341-F and 805-R, but with different probes of 515-P_A, 516-P_B and 515-P_C respectively. 516-P_B supposed to have higher PCR efficiency compared to 515-P_A. 515-P_C was the reverse complementary sequence of 516-P_B. FAM_F4 used 776-F, 1079-R_M, 1079-R_noC, 1079-R_wT and 922-P. 1079-R_M, 1079-R_noC, and 1079-R_wT are similar sequences. The different version of reverse primer was to cater different sequences variant at the locus. 1079-R_noC, and 1079-R_wT added to cover *E*.*faecalis* and *P*.*aeruginosa* respectively. FAM_F5 and 6 used 932-F and 1211R, but with different probes of 1073-P_wC and 1073-P_wT respectively. 1073-P_wC can cover most of the bacterial species. 1073-P_wT were especially designed to cover *P*.*aeruginosa*.

**Table 1:** Primer-probe sets used in this study and their target amplification sequences.

*Target (gene)*	*Amplicon size (bp)*	*Set name*	*Primer and* *probe identity*^a^	*Primer and probe*^b^ *sequence 5’-3’*	*Source*
*General bacteria (DH5α) (16s rRNA)*	*465*	*FAM_F1, FAM_F2, FAM_F3*	*B341-F*	*CCTACGGGAGGCAGCAG*	*[27, 29]*
			*805-R*	*GACTACCAGGGTATCTAATCCTGTT*	*[30, 31]*
			*515-P_A*	*FAM-GTGCCAGCAGCCGCGGTAA-BHQ1*	*Modified from [32, 33]*
			*516-P_B*	*FAM-TGCCAGCAGCCGCGGTAATAC-BHQ1*	
			*515-P_C*	*FAM-CGTATTACCGCGGCTGCTGGCAC-BHQ1*	*[30, 31]*
	*326*	*FAM_F4*	*776-F*	*GGATTAGATACCCTGGTAGTCCA*	*[31, 34]*
			*1079-R_M*	*GCTCGTTGCGGGACTTAAC*	*In this study*
			*1079-R_noC*	*CGCTCGTTGCGGACTTAAC*	
			*1079-R_wT*	*CGCTCGTTACGGGACTTAAC*	
			*922-P*	*FAM-CGCACAAGCGGTGGAGCATGTG-BHQ1*	
	*300*	*FAM_F5, FAM_F6*	*932-F*	*GTGGAGCATGTGGTTTAATTCG*	*In this study*
			*1211R*	*CATTGTAGCACGTGTGTAGCC*	*[33, 35]*
			*1073-P_wC*	*FAM-TGTTGGGTTAAGTCCCGCAACG-BHQ1*	*In this study*
			*1073-P_wT*	*FAM-TGTTGGGTTAAGTCCCGTAACG-BHQ1*	
*Methicillin-resist (MecA)*	*155*	*CY5_C1*	*mecA484-F*	*TGGTATGTGGAAGTTAGATTGGGAT*	*[29, 34]*
			*mecA638-R*	*CTAATCTCATATGTGTTCCTGTATTGGC*	
			*mecA522-P*	*CY5-TTCCAGGAATGCAGAAAGACCAAAGCA-BHQ2*	
	*463*	*CY5_C2*	*MecA-F*	*GTTCCAGATTACAACTTCACCAGG*	*[30, 35]*
			*MecA-R*	*GTGAGGTGCGTTAATATTGCCA*	*In this study*
			*Mec1667-P*	*CY5-TCACCTTGTCCGTAACCTGAATCAGC-BHQ2*	
*S. aureus (Spa)*	*101*	*Spa*	*Spa-F*	*CAGCAAACCATGCAGATGCTA*	*[29, 34]*
			*Spa-R*	*GCTAATGATAATCCACCAAATACAGTTG*	
			*Spa-P*	*TXR-GCTCAAGCATTACCAGAAACTGGTGAAG-BHQ2*	
*Positive control (Lambda dna)*	*200*	*Lambda*	*200bp-F*	*TCGTCGATTTGGTGCCGTAA*	*In this study*
			*200bp-R*	*GCTTCCTGATATGCGAGGCT*	
			*200bp-P*	*HEX-CGCTGGCGGGTTTCCCCTTG-BHQ1*	

^a^ F = forward primer; P = probe; R = reverse primer

^b^ Probe sequence comprises of fluorophore-nucleotides-quencher; FAM stands for Fluorescein; TxR stands for Texas Red; BHQ1,2,3 stands for Black Hole Quencher-1, 2, 3.

#### Spa marker

Similar to the primer/probe design mentioned previously, the forward primer, Taqman probe, and reverse primer are located in the conserved region in a tandem. After downloading the 16s rRNA sequence from NCBI, they were aligned using clustalW multiple alignments with the aid of MEGA7 software [28]. The annealing *T*_*m*_ and the primer quality were checked using fastPCR software, Primer Digital. High linguistic complexity, >80%, and high Primer’s PCR Efficiency, >80%, primers were selected for use. Three sets of 16s primers were designed for this study (see Table 1). FAM_F1, 2 and 3 used B341-F and 805-R, but with different probes of 515-P_A, 516-P_B and 515-P_C respectively. 516-P_B supposed to have higher PCR efficiency compared to 515-P_A. 515-P_C was the reverse complementary sequence of 516-P_B. FAM_F4 used 776-F, 1079-R_M, 1079-R_noC, 1079-R_wT and 922-P. 1079-R_M, 1079-R_noC, and 1079-R_wT are similar sequences. The different version of reverse primer was to cater different sequences variant at the locus. 1079-R_noC, and 1079-R_wT added to cover *E*.*faecalis* and *P*.*aeruginosa* respectively. FAM_F5 and 6 used 932-F and 1211R, but with different probes of 1073-P_wC and 1073-P_wT respectively. 1073-P_wC can cover most of the bacteria species. 1073-P_wT were especially designed to cover *P*.*aeruginosa*.

#### mecA marker

Two sets of primer were investigated for *mecA* gene. The first set was adapted directly from Nakagawa’s work [34]. The second set, MecA-F was modified Stegger *et al*. [34]; MecA-R and Mec1667-P were designed in this study.

#### Lambda marker and template

Bacteriophage lambda (cI857ind 1 *Sam* 7) DNA template was easily available from New England Biolabs (NEB), USA. A 200bp amplicon was designed with the aid of Primer3Plus [36].

### PCR reagent and protocol

The synthesized and modified oligonucleotides were purchased from Bio Basic Canada and Integrated DNA Technologies (IDT). Taqman RTPCR was conducted on CFX96 Touch™ RTPCR Detection System (BioRad, Singapore). PCR amplification was carried out in 20μL reaction mixture containing 1x PCR buffer, 1.344U GoTaq polymerase (Promega, US) and 1pg to 10ng of gDNA. Range of concentrations of primers and probes were tested to optimize refine the protocol. This is discussed in the results and discussion section.

### Development of PCR Protocol

A 2-step protocol was used for TaqMan hydrolysis probe-based runs, as follows: 3 min at 95°C for initial denaturation, followed by 39 cycles of 2 steps consisting of 10s at 95°C for denaturation and 20 sec at 60°C for annealing/extension. PCR running conditions was evaluated across a range of annealing temperatures and running time for each step was decreased gradually. DNA template used was 10 ng of Qiagen-purified DNA from approximately 10^6^ CFU or DNA obtained after thermal lysis for simulant samples, to determine LoD. For singleplex reactions, 0.2 μM of each primer and probe was used while 0.1 μM of each primer and probe was used for multiplex reactions.

### Confirmation of gene amplification signals by AGE

Following signal detection, the amplification products were resolved by agarose gel electrophoresis (AGE) (100 V, 45 min) in 1% agarose, containing 1x GelRed (Biotium, USA) and visualised in UV trans-illuminator (Bio-Rad, USA). The size of the amplified product was compared to DNA ladder (NEB, USA).

### LoD determination for the RT-PCR assay

Ten-fold serial dilutions were performed on the standardized inoculum. For each dilution tested, 10 μL volume of bacterial inoculum was inoculated onto LB agar plates. Colonies were counted after 24 h of incubation at 37°C. At the same time, the same volume was added to blood sample or direct PCR tubes. The lowest bacterial concentration whose RT-PCR gave a consistent *C*_*q*_ and gave a clear band on the agarose gel was recorded as the sensitivity or LoD (Limit of Detection), by plotting the *C*_*q*_ values against each dilution using a regression analysis of the PCR assay.

## Results and discussion

**Fig 1:**
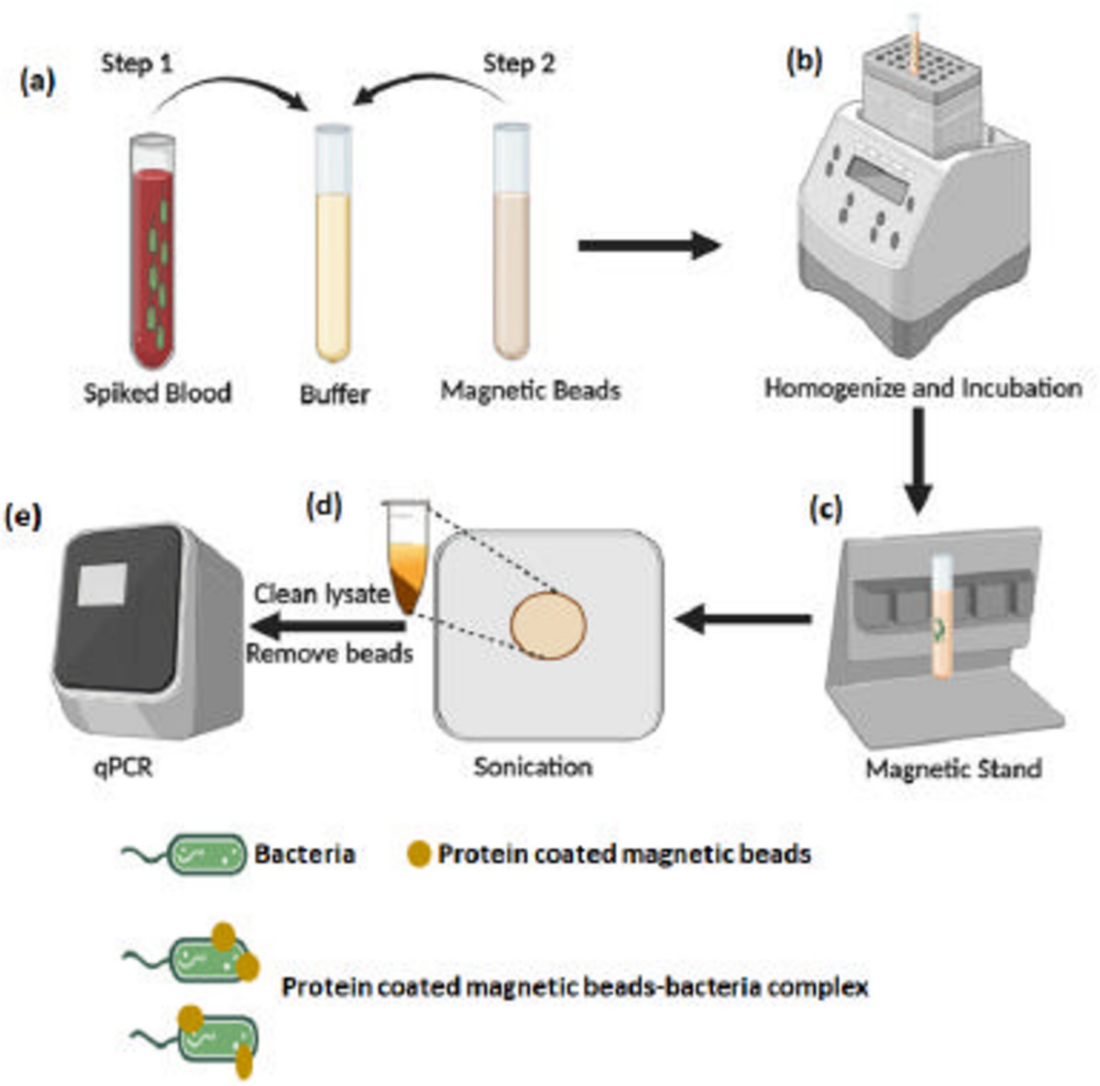
Schematic of the developed protocol to identify MRSA from whole blood showing: **(a)** the addition of equal volumes of spiked blood and buffer to which magnetic beads are added **(b)** the mmixture of spiked blood and ApOH-coated magnetic beads is placed onto a thermomixer maintained at 37ºC, 1000 RPM for 30 mins **(c)** The mixture is placed on a magnetic stand for 10 mins where the beads with the bacteri magnetic beads-bacteria complex are laterally pelleted **(d)** the magnetic beads-bacteria complex are lysed using the in-house sonicator horn **(e)** the clean lysate is added to the PCR reagents on which RT-PCR is performed.

In this study, we designed a multiplex RTPCR assay, to detect the presence of MRSA directly from blood (without a blood culture step), by integrating it with a pre-processing step as shown in the schematic in Fig 1. The pre-processing step involves retrieval of bacteria from blood using protein coated magnetic beads (ApoH-CaptoBAC), followed by in-house sonication for lysis (Fig 1A–1D) and a subsequent PCR step, where the primer/probe system included the detection of general bacteria (16s), SA, *mecA*, and a positive control (Ldna).

### Development of the pre-processing assay

Pre-processing of the blood sample using protein coated magnetic beads followed by lysis resulted in DNA that can be amplified using the developed PCR assay. Pre-processing step is essential to obtain clean lysate that will not hamper or interfere with PCR.

### ApoH-Capto BAC beads kit validation

**Fig 2:**
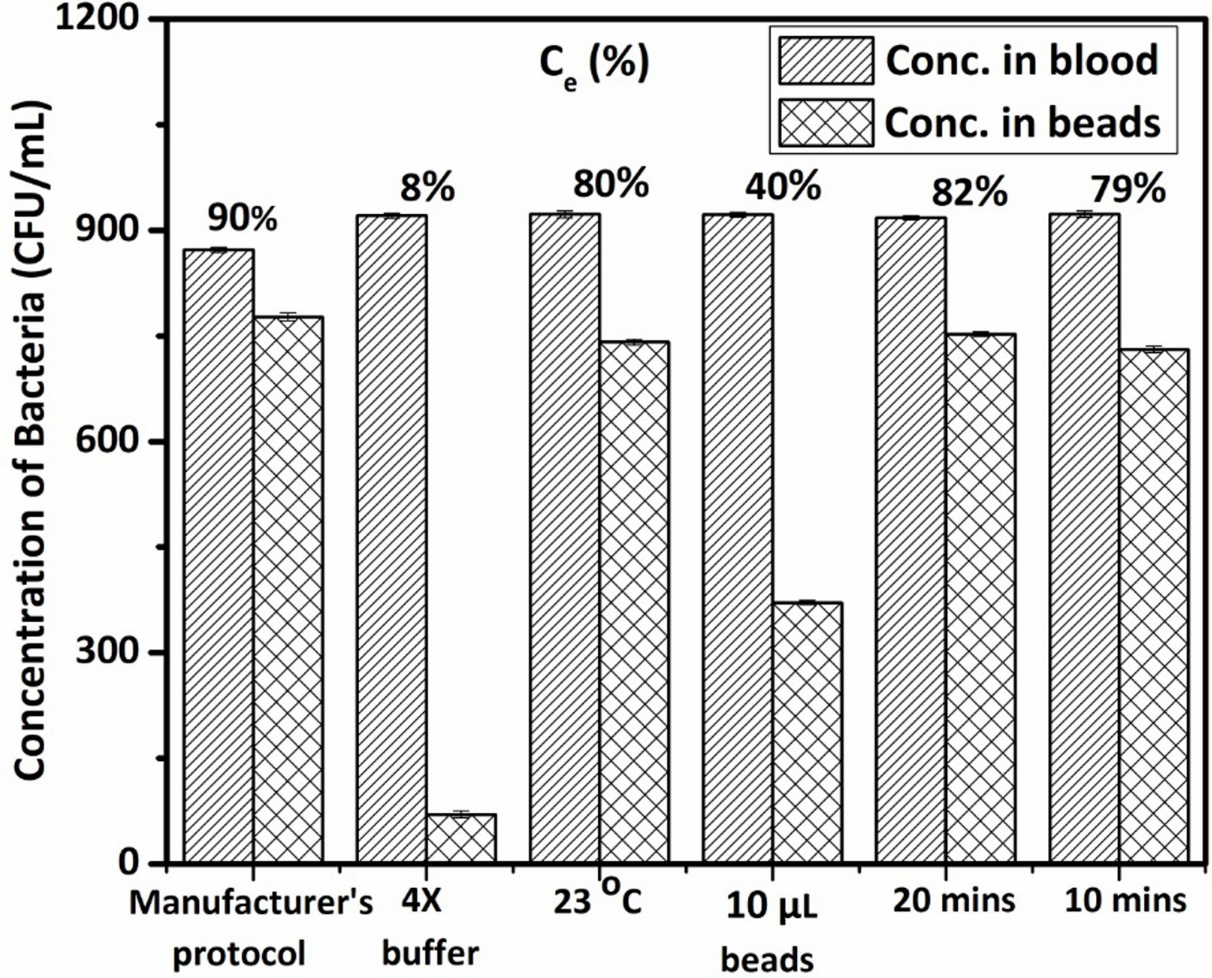
Bar chart showing the concentration of bacteria in spiked blood (bars with right slant lines) along with the concentration of bacteria separated in beads in the form of magnetic beads-bacteria complex (bars with crossed lines) obtained on agar plates after overnight incubation. ∼ 70 μL each of the original spiked blood used for the particular experiment and the collected magnetic beads-bacteria complex after mixing, incubation and magnetic separation were incubated overnight for this analysis. The base manufacturer’s protocol and modifications specified on the X-axis were used to calculate the mentioned captured efficiency (*C*_*e*_).

The manufacturer’s protocol was initially used to test beads’ efficiency to sequester bacteria and the protocol was modified to find the best possible combination of parameters to get maximum isolation of bacteria from blood. Manufacturer’s protocol includes the addition of equal volumes of TTGB buffer, to the collected sample, followed by addition of ApoH beads (Fig 1A) and incubating the mixture for 30 min in a thermomixer (Fig 1B). ApoH beads consists 300 nm magnetic beads coated with activated apolipoprotein, shown to bind to a variety of sepsis causing bacteria, specifically. The 30 min incubation time (at 37°C) with mixing at 1000 rpm enables the contact between magnetic beads and bacteria. Once bacteria bind to the protein coated magnetic beads, they can be isolated from blood and lysed to obtain DNA, the next step. The reaction tube was placed onto a magnetic stand, to enable the lateral pelleting of magnetic beads-bacteria complex, as shown in Fig 1C. The concentration of bacteria in the different solutions (pellet, supernatant blood and initial spiked blood) was then used to calculate the pathogen capture efficiency of the beads (*C*_*e*_) which is the ratio of the concentration of bacteria in the magnetic beads-bacteria complex to the concentration of bacteria in the original blood sample, plotted in Fig 2.

The calculated capture efficiency for the manufacturer’s protocol was ∼90% (as shown in Fig 2) and further modifications to parameters such as buffer concentration, temperature, bead volume, and incubation time were performed to test the effect of these parameters on *C*_*e*_. For example, the buffer concentration was increased to four times the original concentration while the temperature was adjusted to room temperature, and the volume of beads was reduced from 20 μL to 10 μL. Additionally, the mixing and incubation periods were shortened from 30 mins to 20 mins and 10 mins. The *C*_*e*_, graphically depicted in Fig 2, were 8%, 80%, 40%, 82%, and 79% respectively for each modified parameter tested. From the above observation, we concluded that the critical parameters that lead to a drop in *C*_*e*_ (< 75%) cannot be altered in the manufacturer’s protocol and they are buffer concentration (*C*_*e*_ ∼ 8%), reaction volume (*C*_*e*_ ∼ 40%) and concentration of beads in the reaction mixture (*C*_*e*_ ∼ 40%). However, the impact of the other parameters investigated were insignificant. The kit thus enriches microbes from blood in 30 min resulting in a limit of detection (LoD) of 1 CFU/mL from 2–5 mL of whole blood that was calculated using plate count method.

### Sonication

Validation of the sonicator horn used in current work can be found in our previous publication by Zhou *et al*.[25]. The effect of time and temperature due to sonication on the lysis process were examined in our previous study and 30 s was considered the optimal time for complete lysis without damaging the nucleic acids.

### Validation of the RT-PCR assay

The assay focused on the detection of specific bacterial components, including the 16S rRNA gene for bacterium identification, the *Spa* gene for SA identification, and the *mecA* gene for the detection of methicillin resistance. 16S, a common bacterial marker, will help clinicians determine not only MRSA infections but also to confirm other bacterial infections and hence was included in our assay as the positive control. Furthermore, the assay’s true negative was determined by an internal Ldna control. We employed fluorescein amidite (FAM), Texas red (TEXAS), and cyanine 5 (CY5) fluorophores for detecting 16S rRNA, *Spa* and *mecA* genes, respectively. Additionally, HEX was employed for the purpose of internal control (Ldna).

We initially evaluated the PCR running conditions across a range of annealing temperatures and the run time for each step was decreased gradually. The selected temperatures were 55, 60, 65 and 68°C. The extension time was 45s. Primer/probe were able to anneal well up till 68°C. However, Cy5 primer-probe gave observable weaker signal at 65 or 68°C. Hence, for further experiments, 60°C was selected as the annealing/extension temperatures. All primer-probe sets were tested at estimated optimal annealing temperature to check for desired amplifications. Annealing temperature for RT-PCR and concentrations of primers and probes were adjusted to obtain good reaction efficiencies and comparable ***C***_***q***_ values over the entire dynamic range as discussed in the following sections.

**Fig 3:**
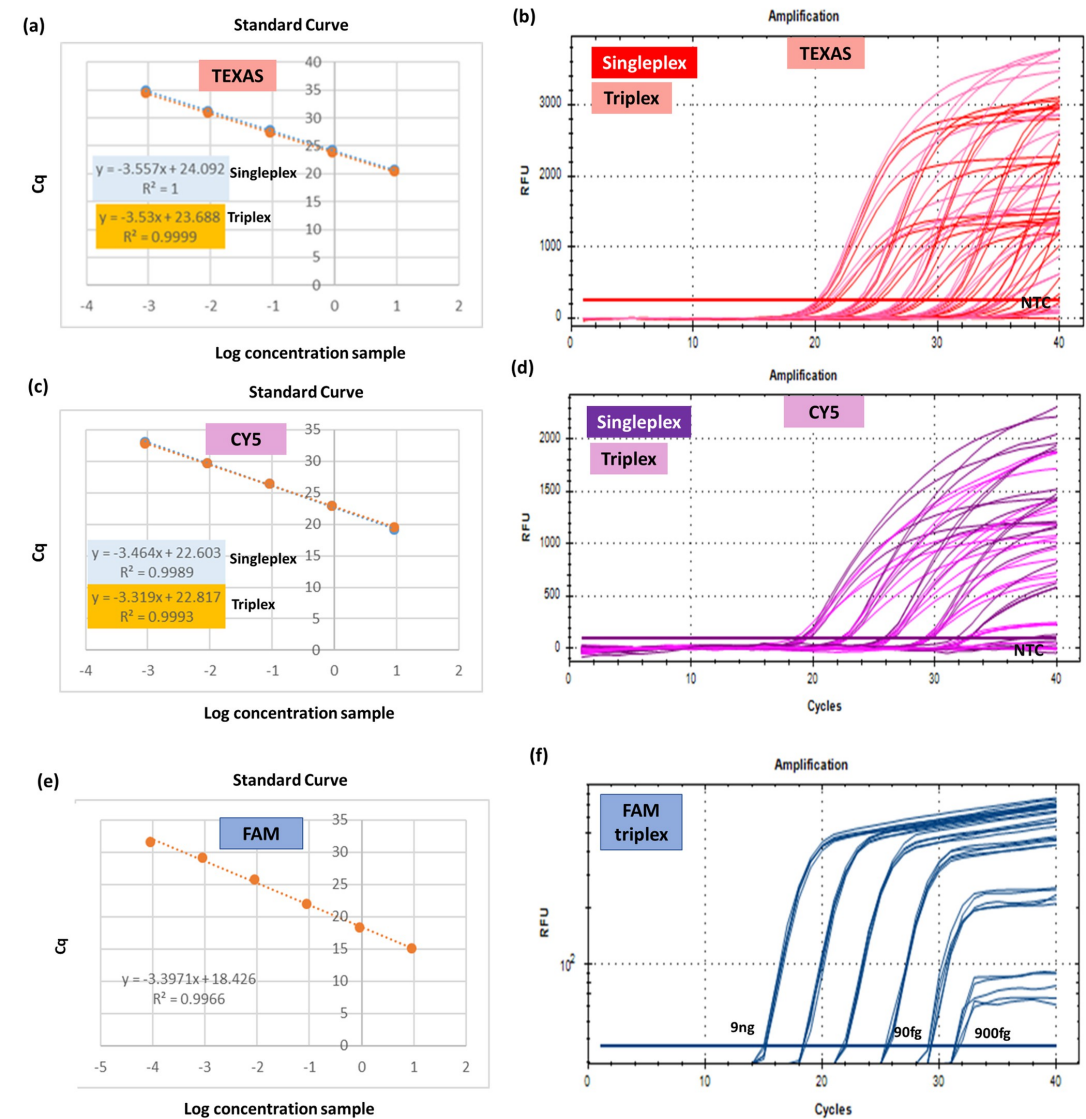
**(a), (c)** and **(e)** show the PCR linearity curves and **(b), (d)** and **(f)** show the PCR amplification curves for **(a)** and **(b)** *spa* marker in the red channel using TEXAS, **(c)** and **(d)** *mecA* marker in the pink channel using CY5 and **(e)** and **(f)** *16s* marker in the blue channel using FAM. All linearity curves shows 90% PCR efficiency and good linearity with R^2^ ∼ 0.99 while the amplification curves showed sigmoidal.

**Fig 4:**
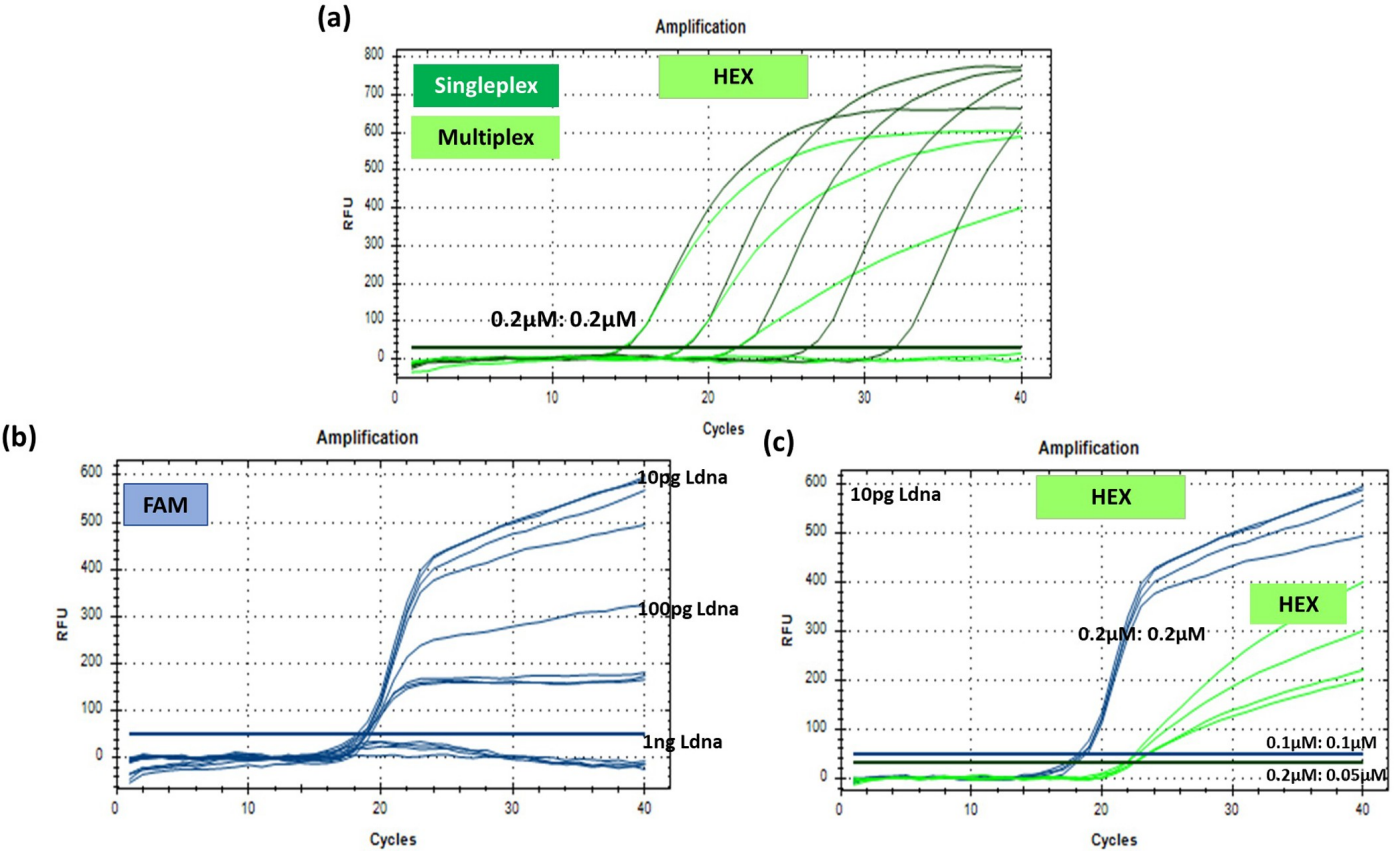
PCR amplification curve **(a)** for the positive internal specificity check with Ldna (lambda DNA) in the green channel using HEX for singleplex and multiplex assays **(b)** for the predetermined concentration of *16s* marker in the blue channel using FAM at different concentration of Ldna and **(c)** for different prime and probe concentration at 10 pg Ldna concentrations for both HEX and FAM.

Assay specificity was evaluated across DNA samples from microorganisms listed in Table 2. Individual singleplex for *Spa*, *mecA*, 16s, and Ldna markers were tested at different primer and probe concentrations followed by triplex/tetraplex assays. The results are presented in Figs 3 and 4 and S1 and S2.

**Table 2:** Performance evaluation of the RT-PCR assay to detect expected markers.

*Sample no*	*Sample content*	*Detected markers*			
		*PC*	*16s*	*spa*	*mecA*
*1*	*Acinetobacter baumannii, ATCC 19606*	+	+	-	-
*2*	*Bacilius subtilis subsp subtis, ATCC 6051*	+	+	-	-
*3*	*Bacteriophage lambda (cI857ind 1 Sam 7)*	+	-	-	-
*4*	*Burkholderia cepacia, ATCC 25416*	+	+	-	-
*5*	*Candida albicans, ATCC 10231*	+	-	-	-
*6*	Candida glabrata, DSM 11226*	+	-	-	-
*7*	Candida parapsiliosis, DSM 5784*	+	-	-	-
*8*	Candida tropicalis, DSM 11953*	+	-	-	-
*9*	Candida krusei, DSM 3433*	+	-	-	-
*10*	*Enterobacter aerogenes, ATCC 13048*	+	+	-	-
*11*	*Enterobacter cloacae, ATCC 13047*	+	+	-	-
*12*	*Enterococcus faecalis, ATCC 29212*	+	+	-	-
*13*	*Enterococcus faecium, ATCC 35667*	+	+	-	-
*14*	*Escherichia coli, ATCC 25922*	+	+	-	-
*15*	*Escherichia coli, DH5alpha*	+	+	-	-
*16*	*Klebsiella oxytoca, DSM 5175*	+	+	-	-
*17*	*Klebsiella pneumoniae, ATCC 13883*	+	+	-	-
*18*	*Morganella morganii, ATCC 25830*	+	+	-	-
*19*	*Proteus mirabilis, ATCC 12453*	+	+	-	-
*20*	*Pseudomonas aeruginosa, ATCC 27853*	+	+	-	-
*21*	*Staphylococcus saprophyticus, ATCC 49453*	+	+	-	-
*22*	*Staphylococcus aureus (MRSA), ATCC BAA-44 (strain HPV107)*	+	+	+	+
*23*	*Staphylococcus aureus subsp aureus, ATCC 29213*	+	+	+	-
*24*	*Staphylococcus aureus subsp aureus, DSM 20231*	+	+	+	-
*25*	Staphylococcus capitis subsp capitius, DSM 20326*	+	+		
*26*	Staphylococcus capitis subsp urealytics, DSM 6717*	+	+		
*27*	*Staphylococcus epidermidis, ATCC 12228*	+	+		
*28*	Staphylococcus haemolyticus, DSM 20263*	+	+		
*29*	Staphylococcus pseudintermeidus, DMS 21284*	+	+		
*30*	Staphylococcus warneri DSM 20316*	+	+		
*31*	*Streptococcus agalactiae (Sero-Group B), ATCC 27956*	+	+		
*32*	*Streptococcus anginosus subso anginosus, DSM 20563*	+	+		
*33*	*Streptococcus dysgalactiae, DSM 20662*	+	+		
*34*	*Streptococcus mutans, ATCC 25175*	+	+		
*35*	*Streptococcus pyogenes, ATCC 19615*	+	+		
*36*	*Streptococcus sanguinis, ATCC 10556*	+	+		
*37*	Streptococcus pneumonia, ATCC 6305*	+	+		

Purified gDNA from *E*. *coli*, MSSA, and MRSA were used to confirm the FAM_F1 to F6 sets of various amplicon sizes listed in Table 1. For FAM_F1 to F3 sets (B341-F, 805-R, 515-P_A, 515-P_B), non-template control (NTC) gave no or low *C*_*q*_ in the late amplification cycles while FAM_F4 to F6 sets, NTC, gave a higher *C*_*q*_. All sets had a strong fluorescence end-point signal, except FAM_F1; the probe with higher PCR efficiency gave a better signal and similar signals were obtained in both the forward and reverse directions. Hence, FAM_F2 and FAM_F 3 were selected for future assay validations and their singleplex (S1 **Fig**) showed good linearity with RFU value ∼1200 to 1400 with *C*_*q*_ value less than 20.

The *spa* marker (TEXAS—red, Figs 3A and 3B and **S2A**) was tested against the organisms listed in Table 2 and were specific to SA and MRSA only. From the S2A Fig, similar *C*_*q*_ values were obtained for all concentration (9 ng to 900 fg) tested in the singleplex assay over the entire linear dynamic range. Good sigmoid amplifications curve for multiplex PCR was obtained (Fig 3B), with robust end-point signal of ∼2200 RFU. The PCR efficiency falls within the acceptable range with slope -3.57 to -3.53 with good linearity (R^2^ ∼1) for both singleplex and triplex assays as shown in Fig 3A.

CY5_C1 and CY5_C2 sets (Table 1) were verified for its detection of *MRSA*, *mecA* marker (CY5—pink, Figs 3C and 3D and S2B) and gave good sigmoid amplifications curve (Fig 3D), good linearity with R^2^ ∼ 0.99 for singleplex as well as triplex (Fig 3C). Comparable percentage of efficiency falls within acceptable range of 90–110% and similar *C*_*q*_ values were observed for the entire linear dynamic range of concentration of 9 ng to 900 fg for singleplex assays as shown in S2B Fig. NTC showed no amplification. However, fluorescent signals were not very strong in the CY5_C1 RT-PCR. To avoid adverse results in the later experiments, CY5_C2 was selected for future assay validation.

For the case of FAM triplex, as can be seen in Fig 3E, 90% PCR efficiency and good linearity were attained in all sets showing the expected sigmoid amplification curve over the dynamic range of concentration from 9 ng to 900 fg. According to the standard linearity curve in Fig 3F, the efficiency was within the acceptable range with a slope of -3.39 and good linearity was obtained with an R^2^ value greater than 0.99.

In addition to the above, internal assay, Ldna (HEX–green, Fig 4A), the positive control helps to confirm cases of true negative. Robust end-point signal was observed at ∼600 and ∼ 700 RFU for both singleplex and multiplex assays, with optimum primer and probe concentration of 0.2μM:0.2μM respectively. The concentration of the primers and probe, and Ldna template were validated by using 5- and 10-fold serial dilutions from 1 ng (2×10^7^ copies) to 100 fg (2×10^3^ copies), by keeping *MRSA* (900 fg) and other markers concentration (FAM, TEXAS, CY5) constant (S1 and S2 Figs). No amplification for multiplex was observed at low template amounts of 1 pg, 100 pg (Fig 4B) and there was a loss in FAM signal at higher Ldna amounts at ∼1 ng, Fig 4A. Further different concentration prime and probe was used for 10 pg of Ldna and loss of signal obtained at 0.2μM: 0.05μM concentration of prime and probe as shown in the Fig 4C. Hence 0.1μM:0,1 μM concentration of prime and probe was considered for further experiments. Table 3 list of final concentrations for the different primer probes combinations for the multiplex assay and its performance is provided in the following section.

**Table 3:** Primers’ and probes’ concentrations for optimised multiplex assay.

Target	Channel	Conc. of each primer (μM)	Conc. of probe (μM)
16s	FAM	0.2	0.2
*Spa*	TEXAS	0.1	0.1
*mecA*	CY5	0.1	0.075
*Lamda dna (Ldna)*	HEX	0.1	0.1

#### AGE analysis

**Fig 5:**
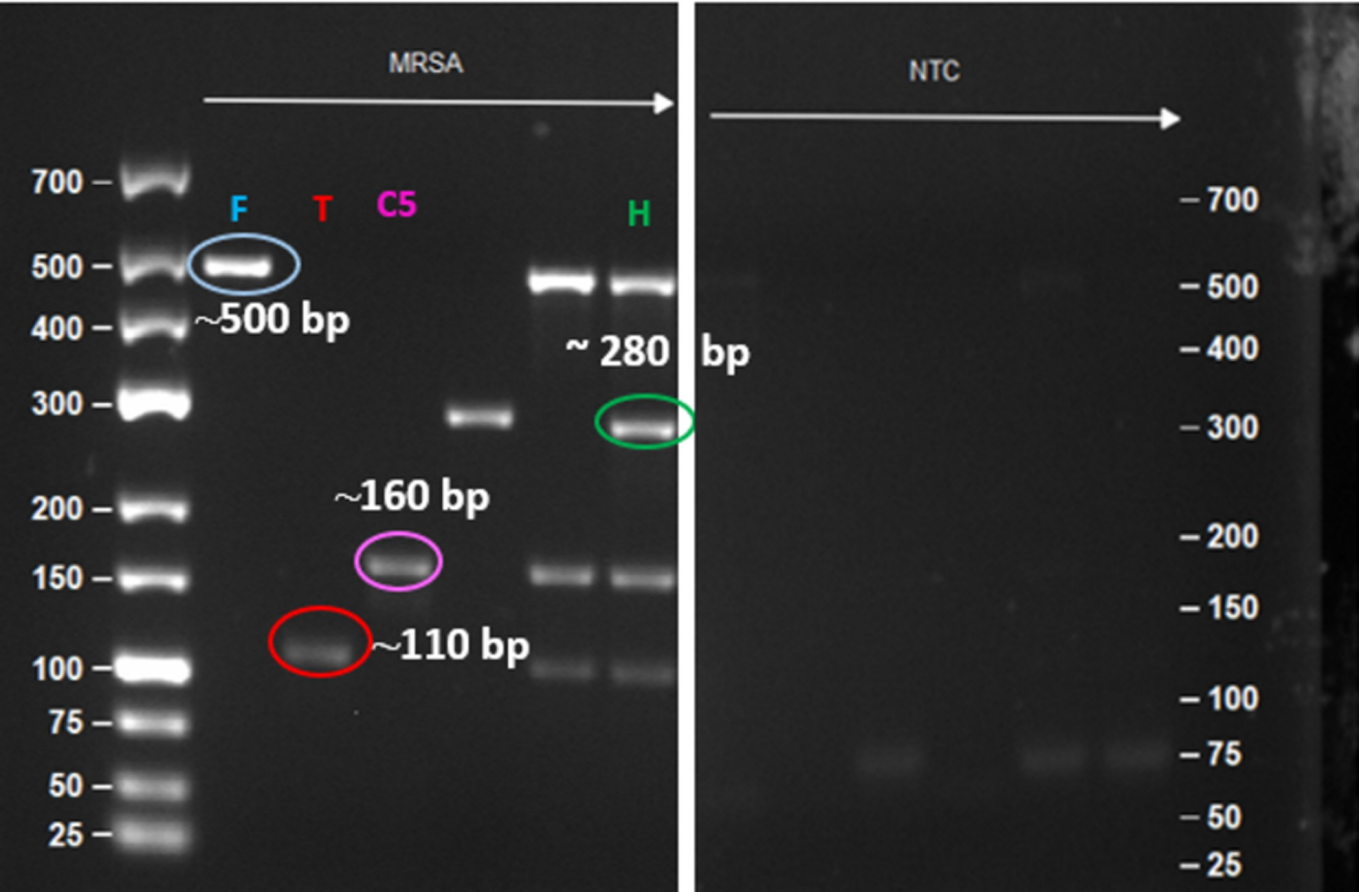
Gel image of the triplex reaction showing bands at ∼500 bp for FAM (marked F with blue circle), ∼110 bp for TEXAS (marked T with red circle) and ∼160 bp for CY5 (marked C5, pink circle) and the corresponding NTC.

AGE (agarose gel electrophoresis; Fig 5) showed that the amplified products were of desired band size for FAM (marked F with blue circle, 500 bp), with no artifacts. The absence of detectable bands in NTC suggested that no contamination or non-specific amplification occurred as also shown in Fig 3C. Fig 5 AGE data shows a weak band at approximately 110 bp (referred to as T, red circle), CY5 (referred as C5) gave a band of 160 bp (pink circle) while HEX (H) gave a band at 280 bp for Ldna, (green circle) for MRSA respectively in AGE analysis. However, the intensity for the FAM marker was high as compared to CY5 and TEXAS for the triplex gel image as shown in **S3 Fig**.

### Performance of multiplex Taqman assay

The PCR assays obtained good sensitivity and good linearity over the range 9 ng to 900 fg of gDNA as shown in Fig 3A, 3C and 3E for *spa*, *mecA* and *16S* markers respectively. For internal assay control, 0.1μM of each primer and probe and 10 pg of Ldna per 20 μL reaction was used to give a positive signal in the absence of target bacterial template as shown in Fig 4C. The new multiplex assay correctly identified and differentiated *MRSA* strains (n = 1), *MSSA* strains (n = 9) and other bacterial strains (listed in Table 2) used in this study as illustrated in Fig 3B, 3D and 3F. No cross contamination and reactions in amplification have been observed in mono-microbial models (as shown in **S4 Fig**) and addition of positive internal control to multiplex reaction was performed to confirm that on-bacterial species did not produce any amplification products. The final concentrations of the primers and probes for different targets for further testing are listed in Table 3.

### Integrated assay LoD for MRSA identification from blood

**Fig 6:**
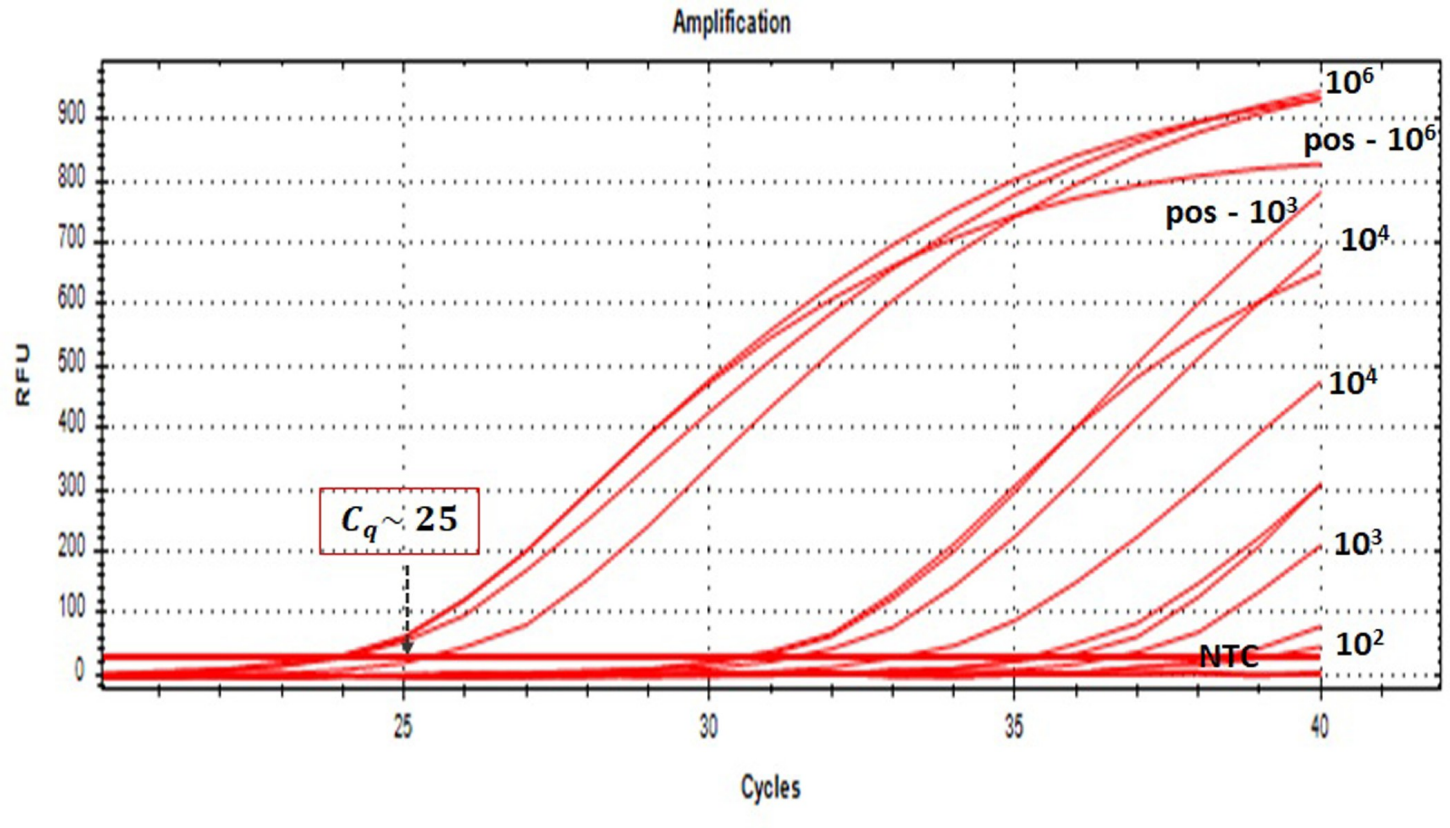
Amplification curve showing the extraction triplicates listed in Table 4 marked using the initial concentration of SA spiked in whole blood.

The novel component of this work, apart from the multiplex PCR assay is the integration of the pre-processing step with the multiplex PCR detection step. The pre-processing step enhances the separation efficiency as well as reduces the time and steps involved in the isolation and separation of pathogen from blood as compared to the standard blood culture technique and other NAT based methods. We performed the integrated protocol listed in Fig 1 wherein the ApoH-CaptoBAC kit was used for the isolation of SA of various concentrations spiked in whole blood (10^7^ to 10^1^ CFU/mL) in 1 hr and then lysed using the sonicator horn and RT-PCR was run on the purified lysate. The *C*_*q*_ values obtained for individual samples performed in triplicates along with their standard deviations, positive controls and NTC are presented in Table 4 while the amplification curve is presented in Fig 6.

**Table 4:** The *C*_*q*_ and standard deviations (SD) for full protocol on whole blood spiked with SA of varying initial concentrations.

Initial concentration of SA in spiked blood (CFU/mL)	Concentration of SA in 70 μL lysate (CFU/mL)	Concentration of SA used for PCR (8 μL of lysate) (CFU/mL)	*C*_*q*_avg	SD (n = 3)
1×10^6^	7.10×10^5^	8.1×10^4^	24.4	0.8
1×10^4^	7.50×10^3^	0.9×10^3^	31.7	1.2
1×10^3^	1.20×10^3^	1.4×10^2^	35.4	0.2
8×10^2^	4.40×10^2^	50	38	1.3
1×10^6^ (positive control)			23.87	
1×10^3^(positive control)			30.45	
NTC			N/A	

The *C*_*q*_ values obtained is used to determine the sensitivity (also called the limit of detection, LoD) which typically refers to the smallest quantity of analyte in a sample that can be detected with a certain probability, even though this amount is not always measured as a precise value. *C*_*q*_ values ≤ 35 are typically considered for quantification of template and as shown in the Table 4, *C*_*q*_ values ≤ 35 are obtained for samples with initial concentration of bacteria in the blood ≥ 1×10^3^ CFU/mL, which is the LoD of the currently developed full-protocol. This value is greater than that obtained for positive control with the same bacterial concentration.

Fig 2 shows that the separation efficiency of the magnetic beads was ∼ 90% with the LoD of the separation step being 1–2 CFU/mL in 2–5 mL of blood However, when the same initial soiked blood sample was considered for further lysis and PCR steps, the LoD of the assay dropped by 2 orders of magnitude. There are two main factors contributing to this decrease and they are: 1. The decrease in the concentration of SA from the isolation to lysis step, ∼ one order of magnitude decrease. The separation step alone shows very good LoD though the process of isolation and lysis using the magnetic stand and sonicator horn has decreased the LoD by 10X. This can be attributed to the cleaning and transfer steps as well as to the non-specific adherence of the NA onto the sonicator horn walls. 2. The decrease in the concentration of SA used for PCR step. The lysate after sonication and purification steps leads to a 70 μL sample. However, the PCR liquid master mix used in the developed assay requires only ∼ 8 μL of this lysate. This further reduces the LoD of the assay by another order of magniture (10X reduction). The reduction in sample volume was not an option in the lysis step due to the lysis chamber volume and the wash requirements which subsequently led to low concentration of NA template in the PCR mix. The two steps above led to a drastic drop in LoD of the full-protocol which was otherwise a robust assay independently which is shown by the close extraction triplicates i.e. the standard deviations (SD) between them are very low of the order of ∼1 for all samples tested. This signifies that the extraction protocol is robust and reproducible.

In order to overcome the above issues and to improve the LoD of the assay, we are currently working on improving the pre-processing step as well as using lyophilized mastermix which can use the full lysate volume after NA extraction. Additionally, this technique is still under development to identify the *CoNs* marker and distinguish between the *MRSA* and *CoNS* strains using the pentaplex assay.

## Conclusions

In this work, we presented a novel multiplex PCR assay that fulfils the requirement for a highly specific method to identify MRSA. Additionally, we incorporated a pre-processing phase to improve the quality of the DNA sample for MRSA identification. Therefore, our study employed a dual strategy, consisting of the utilization of protein-coated magnetic beads to selectively capture pathogens from whole blood, followed by their lysis using a sonicator horn. Subsequently, the processed samples were subject to the developed multiplex Taqman PCR assay for the rapid detection of MRSA. The primer-probe sets used to detect the presence of markers for general bacteria (*16S* rRNA), *S*. *aureus* (*spa*), *mecA*-encoded staphylococcal methicillin resistance associated with PBP2a (*mecA*), and an assay positive control (lambda phage DNA) were incorporated in the study. This study represented one of the initial attempts to explore the feasibility of conducting direct PCR detection using blood samples that have been artificially contaminated, without the prerequisite of a blood culture procedure.

Development of a rapid, sensitive and specific multiplex RT-PCR assay described in this study offers immense potential for routine diagnosis of MRSA and other sepsis-causing pathogens. However, the assay is still under development: high sensitivity and specificity obtained in preliminary studies suggests the future applicability of the optimised assay for direct detection of 3 key genes from patient’s blood samples, with an additional assay control to rule out false negatives.

## Supporting information

S1 Fig PCR Amplification curve for 16s marker: Singleplex FAM. 16s marker: FAM singleplex.
(DOCX)

S2 Fig PCR amplification curve over the entire linear dynamic range of concentration of 9ng to 900 fg for (a) spa marker in the red channel using TEXAS (b) mecA marker in the pink channel using CY5. PCR amplification curve for spa and mec markers.
(DOCX)

S3 Fig Gel image of triplex reaction (F: FAM, C5: Cy5, T: Texas red). AGE data for triplex reaction.
(DOCX)

S4 Fig Specificity check for cross reaction and contamination (a) FAM (b) TEXAS (c) CY5 (d) HEX and (e) Candida. Specificity check for the multiplex assay.
(DOCX)

S1 Raw images (PDF)
