# Supplementary material for: A multiplex Taqman PCR assay for MRSA detection from whole blood
Source: PLoS One. 2023 Nov 27;18(11):e0294782. doi: 10.1371/journal.pone.0294782 (PMC10681265; doi:10.1371/journal.pone.0294782)
Supplement: S2 Fig — PCR amplification curve over the entire linear dynamic range of concentration of 9ng to 900 fg for (a) spa marker in the red channel using TEXAS (b) mecA marker in the pink channel using CY5. PCR amplification curve for spa and mec markers. (DOCX) [file pone.0294782.s002.docx]

***Supporting Information -*** **PCR amplification curve for s*pa* and *mec* markers**

***A multiplex Taqman PCR assay for MRSA detection from whole blood***

Suhanya Duraiswamy^1^^*^, Sushama Agarwalla^1^, Lok Khoi Sheng^2^, Tse Yee Yung^2^, Ruige Wu^2*,^ Zhiping Wang^2^

^1^Department of Chemical Engineering, Indian Institute of Technology Hyderabad, Telangana, 502285, India.

^2^Singapore Institute of Manufacturing Technology (SIMTech), Agency for Science, Technology and Research (A*STAR), 2 Fusionopolis Way, Singapore 138634, Republic of Singapore.

E-mail: [suhanya@che.iith.ac.in](about:blank); [rgwu@simtech.a-star.edu.sg](mailto:rgwu@simtech.a-star.edu.sg)


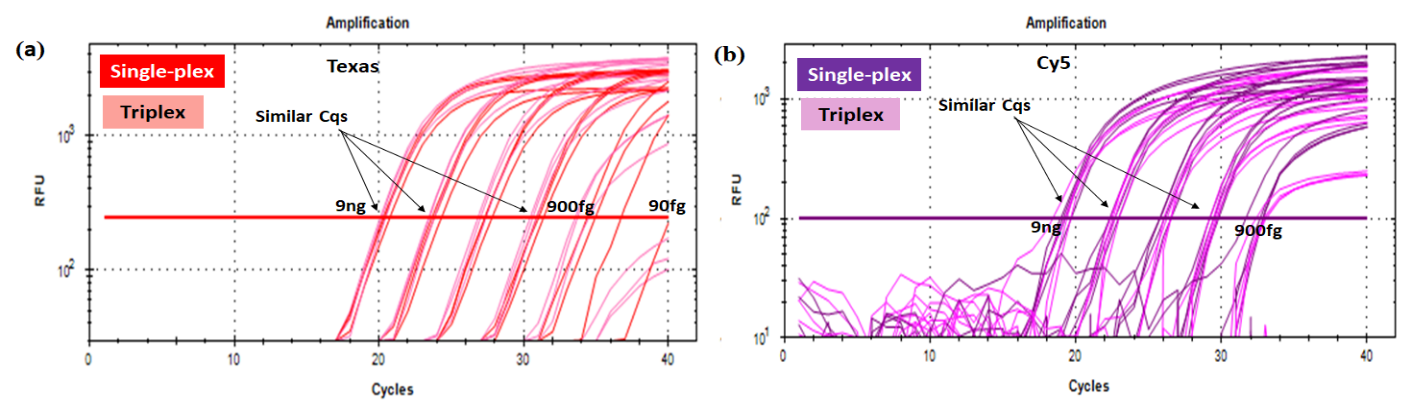


**Fig S2.** PCR amplification curve over the entire linear dynamic range of concentration of 9ng to 900 fg for **(a)** *spa* marker in the red channel using TEXAS **(b)** *mecA* marker in the pink channel using CY5.
