# Supplementary material for: A multiplex Taqman PCR assay for MRSA detection from whole blood
Source: PLoS One. 2023 Nov 27;18(11):e0294782. doi: 10.1371/journal.pone.0294782 (PMC10681265; doi:10.1371/journal.pone.0294782)
Supplement: S3 Fig — AGE data for triplex reaction. (DOCX) [file pone.0294782.s003.docx]

***Supporting Information -*** **AGE data for triplex reaction**

***A multiplex Taqman PCR assay for MRSA detection from whole blood***

Suhanya Duraiswamy^1^^*^, Sushama Agarwalla^1^, Lok Khoi Sheng^2^, Tse Yee Yung^2^, Ruige Wu^2*,^ Zhiping Wang^2^

^1^Department of Chemical Engineering, Indian Institute of Technology Hyderabad, Telangana, 502285, India.

^2^Singapore Institute of Manufacturing Technology (SIMTech), Agency for Science, Technology and Research (A*STAR), 2 Fusionopolis Way, Singapore 138634, Republic of Singapore.

E-mail: [suhanya@che.iith.ac.in](about:blank); [rgwu@simtech.a-star.edu.sg](mailto:rgwu@simtech.a-star.edu.sg)


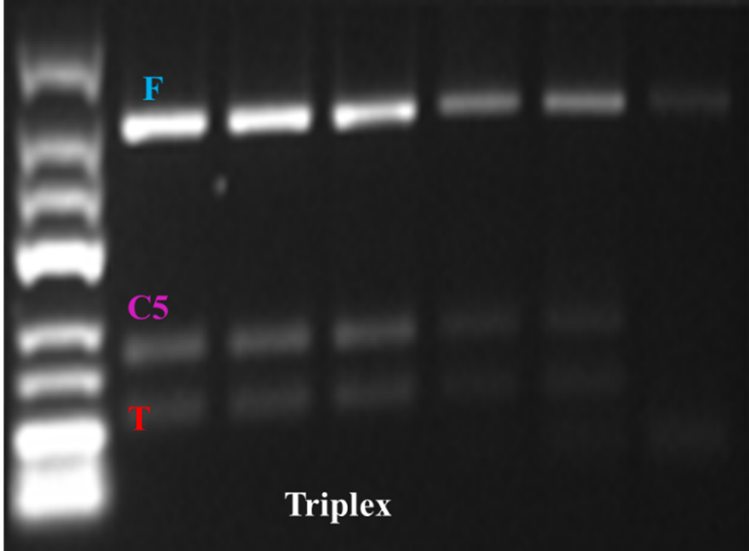


**Fig S3.** Gel image of triplex reaction (F: FAM, C5: Cy5, T: Texas red)
