# Supplementary material for: A multiplex Taqman PCR assay for MRSA detection from whole blood
Source: PLoS One. 2023 Nov 27;18(11):e0294782. doi: 10.1371/journal.pone.0294782 (PMC10681265; doi:10.1371/journal.pone.0294782)
Supplement: S4 Fig — Specificity check for cross reaction and contamination (a) FAM (b) TEXAS (c) CY5 (d) HEX and (e) Candida. Specificity check for the multiplex assay. (DOCX) [file pone.0294782.s004.docx]

***Supporting Information -*** **Specificity check for the multiplex assay**

***A multiplex Taqman PCR assay for MRSA detection from whole blood***

Suhanya Duraiswamy^1*^, Sushama Agarwalla^1^, Lok Khoi Sheng^2^, Tse Yee Yung^2^, Ruige Wu^2*,^ Zhiping Wang^2^

^1^Department of Chemical Engineering, Indian Institute of Technology Hyderabad, Telangana, 502285, India.

^2^Singapore Institute of Manufacturing Technology (SIMTech), Agency for Science, Technology and Research (A*STAR), 2 Fusionopolis Way, Singapore 138634, Republic of Singapore.

E-mail: [suhanya@che.iith.ac.in](about:blank); [rgwu@simtech.a-star.edu.sg](mailto:rgwu@simtech.a-star.edu.sg)

**
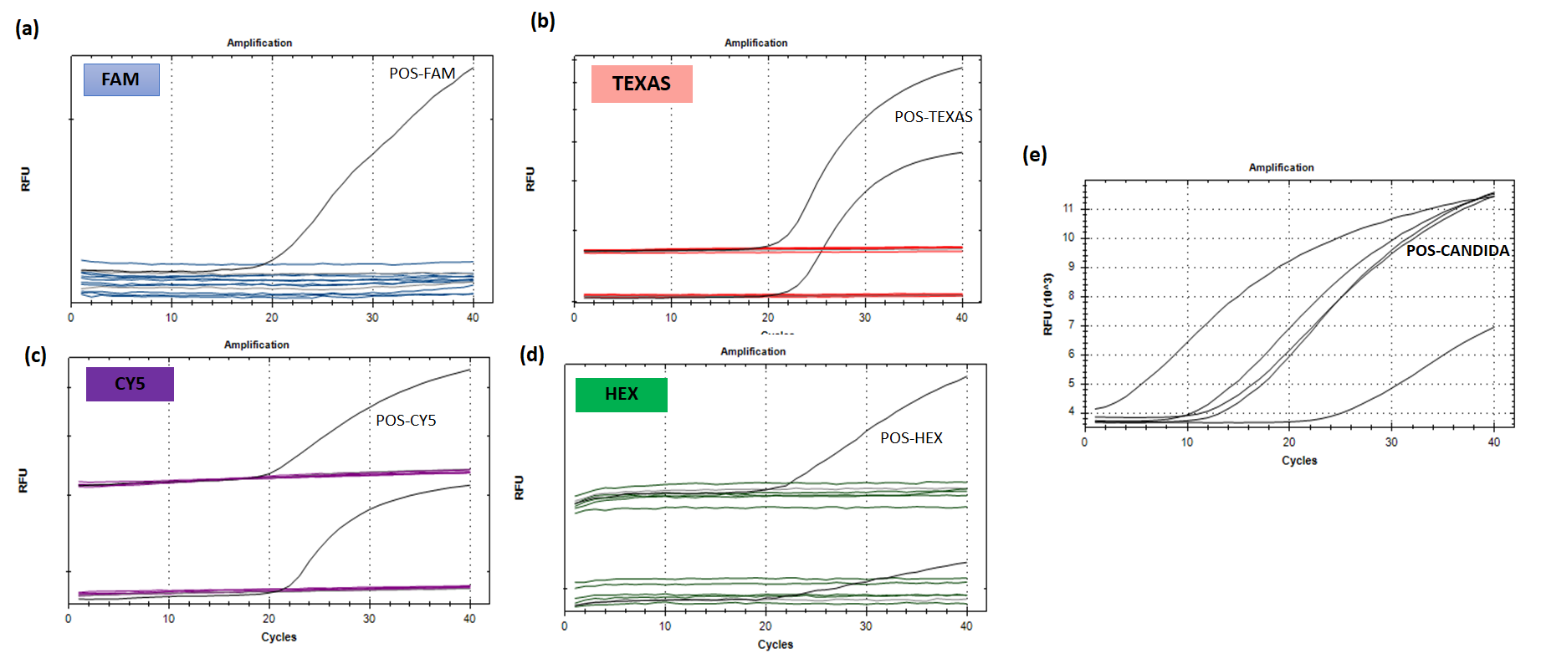
**

**Fig S4.** Specificity check for cross reaction and contamination (a) FAM (b) TEXAS (c) CY5 (d) HEX and (e) Candida
