# Supplementary material for: A multiplex Taqman PCR assay for MRSA detection from whole blood
Source: PLoS One. 2023 Nov 27;18(11):e0294782. doi: 10.1371/journal.pone.0294782 (PMC10681265; doi:10.1371/journal.pone.0294782)
Supplement: S1 Raw images — (PDF) [file pone.0294782.s005.pdf]

Supporting Information - Original Gel images:

A multiplex Taqman PCR assay for MRSA detection from whole blood

Suhanya Duraiswamy[1*], Sushama Agarwalla[1], Lok Khoi Sheng[2], Tse Yee Yung[2], Ruige Wu[2*,] Zhiping Wang[2]

[1]Department of Chemical Engineering, Indian Institute of Technology Hyderabad, Telangana, 502285, India.

[2]Singapore Institute of Manufacturing Technology (SIMTech), Agency for Science, Technology and Research (A*STAR), 2 Fusionopolis Way, Singapore 138634, Republic of Singapore.

E-mail: suhanya@che.iith.ac.in; rgwu@simtech.a-star.edu.sg

Image corresponding to Fig 5:

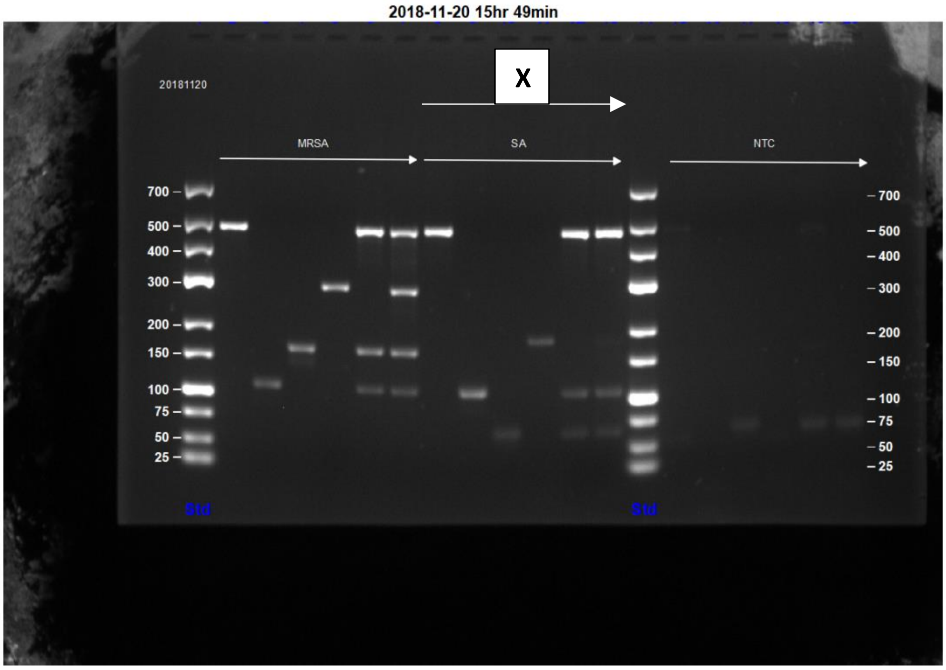

Gel image of the triplex reaction showing 6 lanes for MRSA, followed by 6 lanes for SA and 6 for NTC. The order of the markers are FAM, TEXAS, CY5, 2 lanes of other markers not discussed in this manuscript and HEX. The SA markers are not discussed in this manuscript and have been removed from the Fig 5.

Image corresponding to Fig S3:

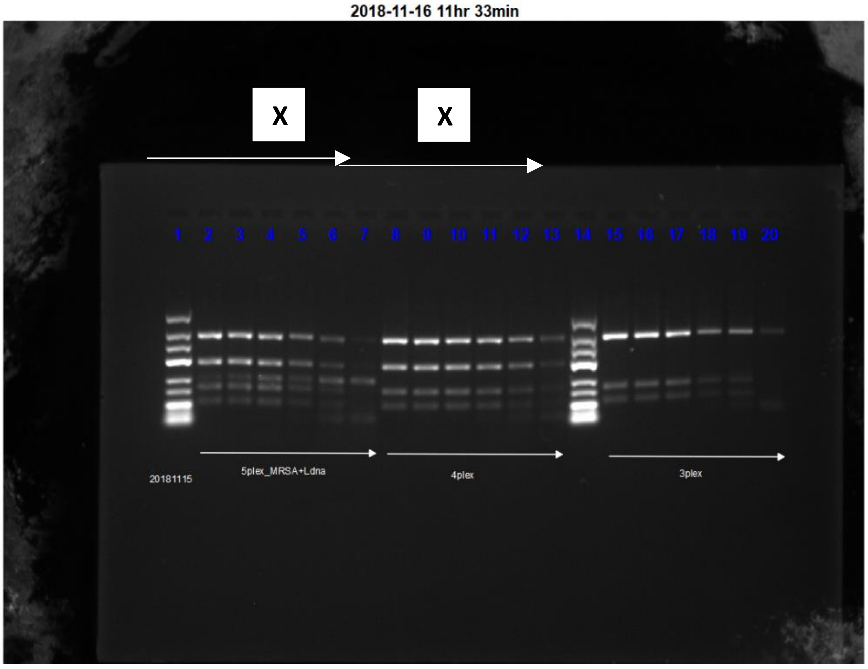

Gel image to determine the primer probe optimal concentrations for the assay. The image shows 6 lanes for pentaplex (not discussed in the manuscript), followed by 6 lanes for tetraplex and 6 for triplex assays. The different concentrations in the different channels labelled 1 to 20 (shown in blue) are 1- ladder, 2,8,15 - 10 ng, 3, 9, 16 - 1ng, 4, 10, 17 - 100 pg, 5, 11, 18 - 10 pg, 6, 12, 19 - 1 pg and 7, 13, 20 - 100 fg of the different markers were tested. The supplement contains only the triplex assay.
